# Comprehensive analysis of the endothelin system in the kidneys of mice, rats, and humans

**DOI:** 10.1042/BSR20240768

**Published:** 2024-07-12

**Authors:** Margi Patel, Nicholas Harris, Malgorzata Kasztan, Kelly A. Hyndman

**Affiliations:** 1 Department of Medicine, Division of Nephrology, Section of Cardio-Renal Physiology and Medicine, University of Alabama at Birmingham, Birmingham, AL 35233, U.K.; 2 Department of Pediatrics, Division of Hematology-Oncology, Section of Cardio-Renal Physiology and Medicine, University of Alabama at Birmingham, Birmingham, AL 35233, U.K.

**Keywords:** chronic kidney disease, Endothelin, kidney, protein, RNA, sex

## Abstract

The intrarenal endothelin (ET) system is an established moderator of kidney physiology and mechanistic contributor to the pathophysiology and progression of chronic kidney disease in humans and rodents. The aim of the present study was to characterize ET system by combining single cell RNA sequencing (scRNA-seq) data with immunolocalization in human and rodent kidneys of both sexes. Using publicly available scRNA-seq data, we assessed sex and kidney disease status (human), age and sex (rats), and diurnal expression (mice) on the kidney ET system expression. In normal human biopsies of both sexes and in rodent kidney samples, the endothelin-converting enzyme-1 (ECE1) and ET-1 were prominent in the glomeruli and endothelium. These data agreed with the scRNA-seq data from these three species, with *ECE1/Ece1* mRNA enriched in the endothelium. However, the *EDN1/Edn1* gene (encodes ET-1) was rarely detected, even though it was immunolocalized within the kidneys, and plasma and urinary ET-1 excretion are easily measured. Within each species, there were some sex-specific differences. For example, in kidney biopsies from living donors, men had a greater glomerular endothelial cell endothelin receptor B (*Ednrb*) compared with women. In mice, females had greater kidney endothelial cell *Ednrb* than male mice. As commercially available antibodies did not work in all species, and RNA expression did not always correlate with protein levels, multiple approaches should be considered to maintain required rigor and reproducibility of the pre- and clinical studies evaluating the intrarenal ET system.

## Introduction

The endothelin (ET) system arose during vertebrate evolution and there is a high degree of similarity at the protein level of these peptides, enzymes, and receptors across species [1,2]. As such, mice and rat models have been instrumental in understanding ET-related mechanisms both in kidney physiology and pathophysiology. We know that under physiological conditions endothelins (ET-1, ET-2, and ET-3) are transcribed as larger precursors that are enzymatically cleaved by endothelin-converting enzyme-1 (ECE1) or ECE2. The active ET-1, ET-2, or ET-3 can then bind to their G protein-coupled receptors, endothelin receptor A (ETA) and/or endothelin receptor B (ETB), to maintain cardiovascular system homeostasis [3]. In the kidney, there is a large amount of ET-1 produced that is necessary for maintaining fluid-electrolyte balance and blood pressure control [4–13]. Under pathophysiological conditions, the ET system can be deranged and can contribute to kidney disease development and progression. In diabetic nephropathy, sickle cell nephropathy [14–17], or hypertensive kidney disease [18,19], excess ET-1 has been recognized as a mediator of kidney hemodynamics alterations, glomerular permeability, and kidney injury. These pre-clinical findings have translated into clinical practice, as recently there have been clinical trials using endothelin receptor antagonists (ERAs). ERAs can slow the loss of glomerular filtration [20], improve proteinuria [21], and improve pain [22] in patients with diabetic nephropathy. In 2023, it was announced that sparsentan (a dual ERA plus angiotensin II receptor blocker), reduced proteinuria in patients with focal segmental glomerular sclerosis (FSGS) [23] or IgA nephropathy [24].

To best understand endothelin-related physiological or disease driving actions, it is important to have a set of tools to interrogate the endothelin system. There are substantial limitations to antibody-based detection systems [25]. Additionally, it has been suggested that monoclonal or non-animal antibodies are more robust and ethical, as they do not require invasive procedures involving animals [26]. However, RNA measures do not reflect enzymatic activity, receptor binding, and do not always correlate with protein abundance [27,28]. Over the past few years, kidney single cell and single nucleus RNA-sequencing technology has provided much insight into the different kidney cell types, and gene networks involved in development, physiology, and kidney disease (e.g., [29–33].). Thus, using comprehensive, multidisciplinary methodological approaches our study aimed to provide rigorous evidence for characterization of renal ET system in mice, rats, and humans regarding age, sex, diurnal expression, or kidney disease status.

## Methods

### Animals, human subjects, and tissue collection

All animal use and welfare adhered to the NIH *Guide for the Care and Use of Laboratory Animals* following a protocol reviewed and approved by the Institutional Laboratory Animal Care and Use Committee of the University of Alabama at Birmingham (UAB). IRB exemption for de-identified human biopsies was also granted by UAB(IRB-151016003). All animal experiments were conducted at UAB unless otherwise noted.

At UAB, male and female Sprague-Dawley rats (10−12 weeks of age) were purchased from (Envigo, Indianapolis, IN). Male and female (10−11 weeks of age) C57BL/6J mice used in the study were from our in-house colony. All rodents were maintained in an animal facility with a 12 h light:12 h dark cycle and fed a standard chow diet with ad lib access to tap water.

The rats and mice were anesthetized with 2% isoflurane and euthanized via bilateral thoracotomy, and the kidneys immediately excised, decapsulated, and cut into either cross section (mice) or coronal section (rats) and immediately fixed in 10% neutral buffered formalin for 24 h. The samples were then washed 1× with 10 mM phosphate buffered saline (PBS) and stored in 70% ethanol in water until dehydrated and embedded in paraffin.

De-identified human kidney biopsy samples, fixed, and embedded in paraffin were acquired from the Cooperative Human Tissue Network (CHTN), and obtained from 5 women (1 autopsy, 4 surgical biopsies), and 4 men (1 autopsy, 3 surgical biopsies). Analysis of all kidney biopsies was completed by a CHTN pathologist and scored as normal. Demographics for these subjects that we had access to are reported in Supplementary Table S1.

### Immunohistochemical analysis of human and rodent kidneys

All kidneys were sectioned at 4 µm and placed on Colorfrost Plus Slides (Fisher Scientific) and underwent immunohistochemical staining as we previously described [34]. Primary antibodies and their concentrations are listed in Table 1. Negative controls were incubated with the antibody diluent of 2.5% normal horse serum (also used as the protein block) instead of a primary antibody. For mouse monoclonal antibodies (antibodies against ECE1, ETA, or ETB) tested on the mouse tissue, the Mouse on Mouse (M.O.M) Immunodetection Kit was used (Vector Labs). Immunoreactivity on all slides was detected using ImmPACT® DAB Substrate Kit, Peroxidase (HRP) for 5 min. The Olympus Bx53 and Dp28 digital camera were used to visualize and image the slides at 40× magnification.

**Table 1:** Primary antibodies tested in the present study (no and yes represent whether the antibody worked in that species)

Antibody	Company	Host	Catalog number	Lot number	Dilution	Human	Rat	Mouse
Big ET1	Phoenix Pharmaceuticals	Rabbit	H-023-12	00095-1	1/2000	No	No	Yes
ECE1 A6	Santa Cruz Biotechnology	Mouse	sc-376017	A2621	1/100	Yes	Yes	Yes
ET1	Phoenix Pharmaceuticals	Rabbit	H023-01	01208-2	1/5000	Yes	No	Yes
ET2	Phoenix Pharmaceuticals	Rabbit	H-023-13	01734-1	1/2000	Yes	No	No
ET3	Phoenix Pharmaceuticals	Rabbit	H-023-17	01291-1	1/5000	No	No	No
ETB C5	Santa Cruz Biotechnology	Mouse	sc-518149	C0124	1/500	No	Yes	No
ETA-F12	Santa Cruz Biotechnology	Mouse	sc-515948	L0721	None worked	No	No	No
ETA-C4	Santa Cruz Biotechnology	Mouse	sc-518120	D0120	None worked	No	No	No
ETB-5H2	Santa Cruz Biotechnology	Mouse	sc-293198	E2022	None worked	No	No	No

### Single cell or nucleus RNA sequencing data and analysis

On June 1, 2022 all publicly available kidney single cell or single nucleus RNA-sequencing data from human, rat, and C57bl/6j mice were downloaded. Cell clustering of the human data obtained from the Kidney Precision Medicine Project (KPMP) data was used as provided [35,36]. The rat and mouse data were re-analyzed to generate *de novo* cluster maps, using the package *Seurat* version 4.1.1.

### Human

The human kidney dataset (.h5Seurat file) was the single cell dataset available from the KPMP and included men and women and 20 samples from 18 living donors (LD) biopsies, 12 acute kidney injury (AKI) biopsies, and 15 chronic kidney disease (CKD) biopsies [36]. The demographics of these subjects that we could access are presented in Supplementary Table S2. Differentially expressed genes (DEGs) were determined using the *FindMarkers* function and the *wilcox* test. Baseline sex differences were determined by comparing LD men versus woman. Additional comparisons within each sex included LD to AKI, LD to CKD, and AKI to CKD. All annotations and cell counts are listed in Supplementary Table S3.

### Rat

The control groups data generated using male and female *Rattus norvegicus*, at 5 months (young) or 27 months (aged) of age rats were obtained from GEO GSE137869 [37]. Count matrixes for the samples were created using the CellRanger output files. Samples were filtered using nFeature_RNA>200 & nFeatureRNA < 3000 & percent.mt < 50 (there were very high mitochondrial genes in these samples). Each sample was normalized using the LogNormalization function, *ScaleData (features = all.genes), FindNeighbors (dims = 1:20), FindClusters (resolution = 1.4)*. Next, the samples were merged, normalized, scaled, and *de novo* clusters were determined. Following batch correction using the *Harmony* the final rat dataset was created. Clusters were annotated based upon manual inspection of known kidney cell type markers as previously published [33,38,39]. DEGs were determined between male and female rats (regardless of age), or effects of age within a sex (e.g., young male compared with old male) using the *FindMarkers* function and DESeq2 test.

### Mice

The mouse dataset was generated from single cell and single nucleus RNA studies on the kidneys collected during daytime (personal communication); GSE157079 [40], GSE180420 [41], GSE182256 [42], GSE164273 [43], GSE129798 [30], GSE193649 [44], GSE139107 [45], and GSE119531 [46]. Additionally, we included our recent ad libitum C57bl6J, male and female, single nucleus RNA sequencing from mice that were euthanized at midnight (GSE232662 [38]). The count matrixes were imported into Seurat and filtered as explained in the rat analyses except *percent.mt < 5*. Each study was normalized using the LogNormalization function, *ScaleData (features = all.genes), FindNeighbors (dims = 1:20), FindClusters (resolution = 1.4)*. Next, all nine studies were merged into one object, and the normalization, scaling, finding neighbors, and clusters were conducted *de novo*. Batch correction using the program *Harmony* was used to create the final mouse dataset. DEGs were determined between midnight and daytime samples (regardless of sex), or the effects of diurnal time within a sex (e.g., female midnight compared with female daytime) using the *FindMarkers* function and the *wilcox* test.

The bulk kidney (GSE232792) and inner medullary RNA-sequencing (GSE195786) datasets were recently published [38,47]. Normalized counts from DESeq2 and adjusted *P*-values are reported.

### Endothelin system genes profiling

Utilizing rat, mouse and human DEGs datasets described above we profiled the kidney ET system including (rodent/human gene annotation): endothelin-1: *Edn1/EDN1, endothelin-2: Edn2/EDN2, endothelin-3: Edn3/EDN3, endothelin A receptor (ETA): Ednra/EDNRA, endothelin B receptor (ETB): Ednrb/EDNRB, endothelin converting enzyme-1: Ece1/ECE1, endothelin converting enzyme-2: Ece2/ECE2*. Genes with an adjusted *P*-value < 0.05 were considered statistically significant. Dot plots using these gene lists were generated using Seurat. Violin plots of differentially expressed genes were generated with Prism (v.10.2.3).

### Plasma and urinary ET-1 excretion

Plasma ET-1 concentrations (pg/ml), urinary ET-1 excretion (ng/day or pg/day/kg body weight) or fractional excretion (FEET-1%) were consolidated from the previously published clinical and pre-clinical model studies. When necessary, units were converted to be the same for ease of comparison among the studies. For the human data mean (standard deviation) is reported, while for the rodents it is mean (standard error of the mean). Plasma ET-1 and ET-1 excretion was not available from the participants or rodents reported in the RNA sequencing experiments.

## Results

### Human

**Figure 1:**
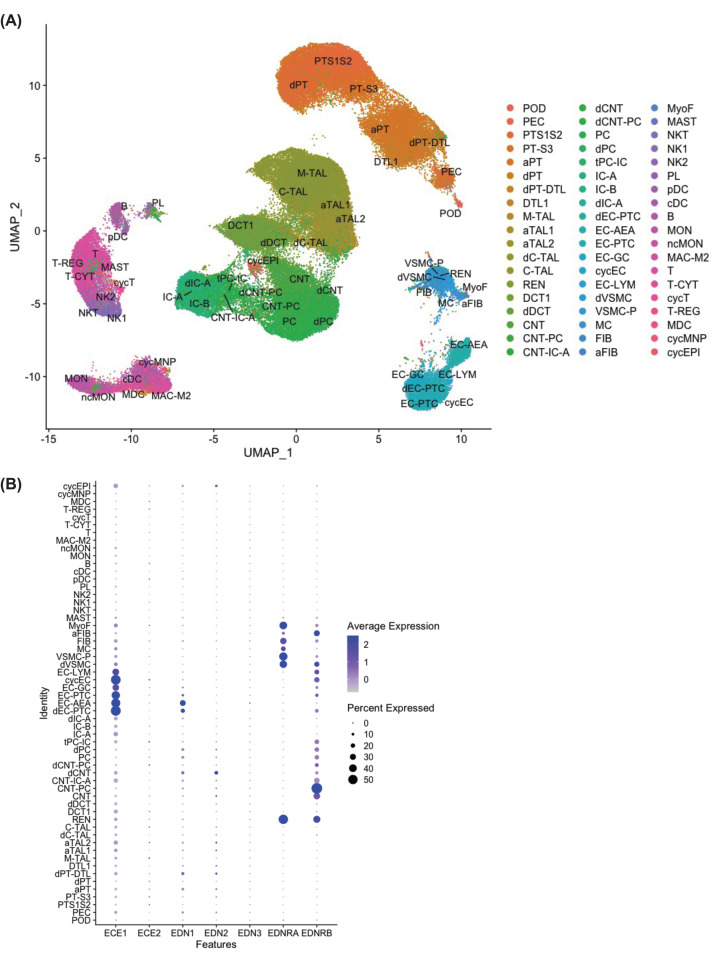
Human kidney single cell analyses from the Kidney Precision Medicine Program [36] (**A**) Unsupervised clustering of the Kidney Precision Medicine Program human kidney single cell RNA-sequencing. A total of 11,0346 cells from living donor controls (*n*=18), chronic kidney disease (*n* =15), and acute kidney injury (*n*=12) subjects are included. (**B**) Dotplot of the RNA expression of the endothelin related genes across the clusters. Abbreviations: aFIB, Fibroblast (adaptive/maladaptive/repairing); aPT, Proximal Tubule Epithelial Cell (adaptive/maladaptive/repairing); aTAL1, Thick Ascending Limb Cell Cluster 1 (adaptive/maladaptive/repairing); aTAL2, Thick Ascending Limb Cell Cluster 2 (adaptive/maladaptive/repairing); B, B cell; C-TAL, Cortical Thick Ascending Limb Cell; cDC, Classical Dendritic Cell; CNT, Connecting Tubule Cell; CNT-IC-A, Connecting Tubule Intercalated Cell Type A; CNT-PC, Connecting Tubule Principal Cell; cycEC, Endothelial Cell (cycling); cycEPI, Epithelial cell (cycling); cycMNP, Mononuclear Phagocyte (cycling); cycT, T cell (cycling); dC-TAL, Cortical Thick Ascending Limb Cell (degenerative); dCNT, Connecting Tubule Cell (degenerative); dCNT-PC, Connecting Tubule cell-Principal cell (degenerative); DCT1, Distal Convoluted Tubule Cell Type 1; dDCT, Distal Convoluted Tubule Cell (degenerative); dEC-PTC, Peritubular Capillary Endothelial Cell (degenerative); dIC-A, Intercalated Cell Type A (degenerative); dPC, Principal cell (degenerative); dPT, Proximal Tubule Epithelial Cell (degenerative); dPT-DTL, Proximal Tubule Epithelial Cell/Descending Thin Limb Cell (degenerative); DTL1, Descending Thin Limb Cell Type 1; dVSMC, Vascular Smooth Muscle Cell (degenerative); EC-AEA, Afferent/Efferent Arteriole Endothelial Cell; EC-GC, Glomerular Capillary Endothelial Cell; EC-LYM, Lymphatic Endothelial Cell; EC-PTC, Peritubular Capillary Endothelial Cell; FIB, Fibroblast; IC-A, Intercalated Cell Type A; IC-B, Intercalated Cell Type B; M-TAL, Medullary Thick Ascending Limb Cell; MAC-M2, M2-Macrophage; MAST, Mast cell; MC, Mesangial Cell; MDC, Monocyte-derived Cell; MON, monocyte; MyoF, Myofibroblast; ncMON, Non-classical Monocyte; NK1, Natural killer 1; NK2, Natural killer 2; NKT, Natural Killer T Cell; PC, Principal cell; pDC, Plasmacytoid Dendritic Cell; PEC, Parietal Epithelial Cell; PL, Plasma Cell; POD, Podocyte; PT-S3, Proximal Tubule Epithelial Cell Segment 3; PTS1S2, Proximal Tubule Epithelial Cell Segment 1&2; REN, Renin-positive Juxtaglomerular Granular Cell; T, T Cell; T-CYT, T cytotoxic; T-REG, T regs; tPC-IC, Principal-Intercalated Cell (transitional); VSMC-P, Vascular Smooth Muscle Cell/Pericyte.

The KPMP dataset consisted of 110,346 cells from the biopsies of LD, CKD, and AKI subjects. The CKD subjects consisted of diabetic kidney disease (DKD) and hypertensive chronic kidney disease subjects (HCKD, Supplementary Table S2). Unsupervised cluster lead to the identification and annotation of 57 clusters of all the expected cell types (e.g., epithelial, vascular, immune cells, Figure 1A, Supplementary Table S3). The KPMP also annotated ‘adaptive’ clusters that represent adaptive/maladaptive/repairing cells, ‘cycling’ those that have increased cell cycle markers, ‘degenerative’ those that have lost differentiation markers, and ‘transitional’ those that have an intermediate genetic signature sharing the same parental lineage [35]. All cell type annotations and definitions can be found in Supplementary Table S3.

In these clusters, we observed enhanced expression of *ECE1* in the endothelial cells, while *ECE2* had very low expression throughout the kidney (Figure 1B). *EDNRA* was highly expressed in vascular smooth muscle cells, fibroblast, mesangial cells, and renin positive cells (Figure 1B). *EDNRB* was highly expressed in the connecting tubule and principal cells (Figure 1B). *EDN1* was highly expressed in the kidney arteriole endothelial cells, while *EDN2* and *EDN3* had low expression (Figure 1B).

**Figure 2:**
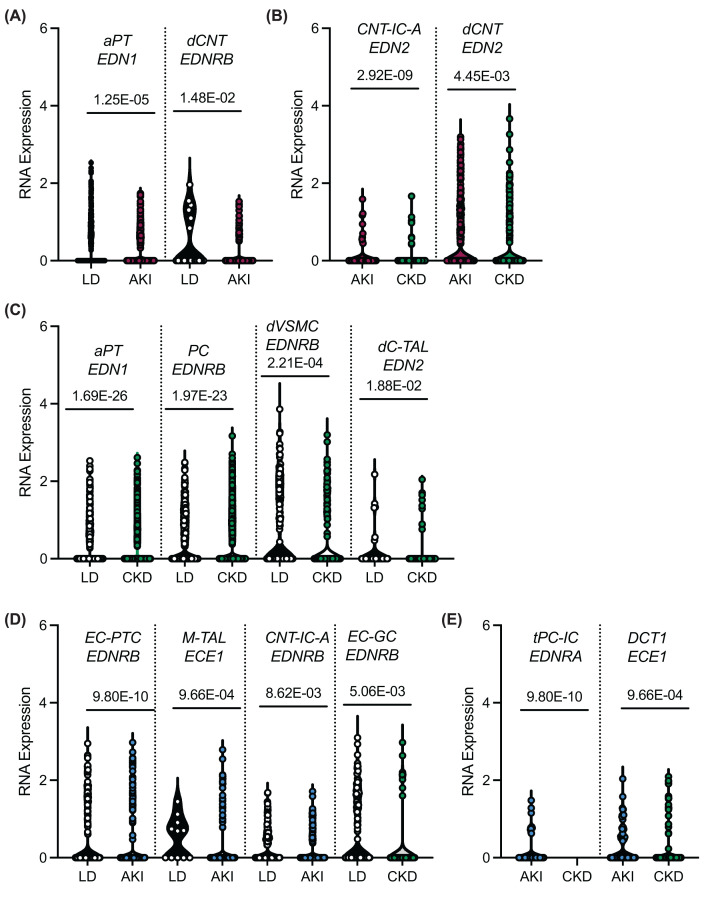
Violin plots with individual kidney cells from human biopsies of the differentially expressed genes related to the endothelin pathway (**A**) Living donor (LD) compared with acute kidney injury (AKI) samples from women, (**B**) AKI compared with chronic kidney disease (CKD) samples from women, (**C**) LD compared with CKD samples from women. (**D**) LD compared with AKI or CKD kidney samples from men, (**E**) AKI compared with CKD samples from men. *P*-values adjusted for multiple comparisons reported. aPT, adaptive proximal tubules; CNT-ICA, connecting tubule intercalated cell alpha; CNT-PC, connecting tubule principal cell; dCNT, degenerative connecting tubule; dCNT-PC, degenerative connecting tubule principal cell; DCT1, distal convoluted tubule-1; dC-TAL, degenerative cortical thick ascending limb; dM-TAL, degenerative medullary thick ascending limb; dVSMC, degenerative vascular smooth muscle cell; EC-GC, glomerular endothelial capillary; EC-PTC, peritubular capillary endothelial cell; M-TAL, medullary thick ascending limb; PC, principal cell; tPC-IC, transitional principal-intercalated cell.

There were only a few statistically significant DEGs between the sexes or within each sex comparing kidney disease status (Figure 2). In samples from LD, men had 2.6 times greater expression of *EDNRB* in the glomerular endothelial cells compared with women (*P* = 4.40E-35), but 30% fewer *EDNRB* transcripts in degenerative connecting tubule principal cells (*P* = 1.51E-02). In women with AKI, *EDN1* transcripts were significantly fewer in adaptive proximal tubules (aPT) and *EDNRB* was fewer in degenerative connecting tubules compared with the LD (Figure 2A). In women with CKD compared with CKD, *EDN2* was significantly lower in the connecting tubule intercalated alpha cells (CNT-IC-A), degenerative connecting tubules (dCNT), and degenerative connecting tubule principal cells (dCNT-PC, Figure 2B). Finally, in different kidney cell types, CKD women had fewer copies of *EDNRB* and *EDN2* than the LD kidneys (Figure 2C). When comparing among the samples from men, LD had 1.5-fold greater expression of *EDNRB* in the peritubular endothelial cells (EC-PTC) and 1.2-fold greater *EDNRB* in the CNT-IC-A compared with these cells from AKI subjects (Figure 2D). *ECE1* in the medullary thick ascending limb (M-TAL) was 1.4-fold higher in the LD than AKI men samples and in the glomerular endothelial cells (EC-GC) from LD had a 1.7-fold greater expression of *EDNRB* compared with CKD samples (Figure 2D). When comparing AKI and CKD samples from men, *EDNRA* was 1.2-fold greater in the transitional principal cells-intercalated cells (tPC-IC) and ECE1 was 1.3-fold greater in the distal tubule 1 (DCT1) *ECE1* (Figure 2E).

**Figure 3:**
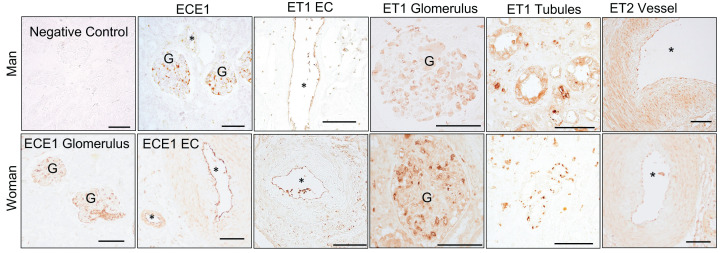
Representative images from human kidney biopsies used for immunolocalization of the ET system The negative control lacked the primary antibody. Endothelin converting enzyme -1 (ECE1), endothelin-1 (ET-1), endothelin-2 (ET2) were abundant in the endothelium (EC) of large kidney blood vessels, and glomeruli (G). ET-1 was also found in some collecting ducts. Asterisks (*) marks blood vessels. Scale bar represents 100 micrometers.

In human kidney biopsies, ET1, ET2, and ECE1 were immunolocalized. Despite of our immense efforts we were unable to find reliable ETA and ETB antibodies that worked with human kidney sections (Table 1). In agreement with our scRNA-seq data, ECE1 was highly abundant in the endothelium of kidney vessels and glomeruli (Figure 2). Likewise, ET1 was abundant in the kidney endothelium including the glomerular endothelium (Figure 3). ET1 was also detected in some of the collecting ducts. We also detected endothelial expression of ET2 intra-renal branched renal arteries and vessels no smaller than the interlobar arteries and not the glomeruli (Figure 3).

Plasma and urinary ET-1 excretion for healthy volunteers, or participants with CKD, hypertension (with and without albuminuria), and diabetes (with and without albuminuria) from other published studies are reported in Table 2. Healthy volunteers had a lower plasma ET-1, and less ET-1 excretion than the participants who had CKD, hypertension, or diabetes (Table 2). One exception was reported by Hwang et al. [48] where their participants who were mildly hypertensive (mean 24 h ambulatory pressure 141 ± 7/91 ± 5 compared with the normal controls 126 ± 8/79 ± 6, *P*<0.05) had similar plasma ET-1 and ET-1 excretion (Table 2). However, participants with hypertension or diabetes with albuminuria had greater plasma ET-1 than participants who did not have albuminuria [49] (Table 2).

**Table 2:** Plasma ET-1, urinary ET-1 excretion, and fractional excretion from different clinical cohorts (Mean [SD])

Study Participants	Sex	Age	*n*	Plasma ET-1, pg/ml	Urinary ET-1 Excretion	Excretion units	FEET-1%	Reference
Healthy volunteers	Men	35(8)	11	n.r.	3.4 (1.3)	pg/day/kg b.w.		Gohar et al. [51]
	Women	33(10)	12	n.r.	6.0 (3.1)^*^	pg/day/kg b.w.		
Healthy volunteers	both	31(10)	10	1.2 (0.4)	1.25 (0.60)	ng/day		Hunter et al. [85]
Non-CKD	both	48 (9)	27	4.6 (1.0)	n.r.		1.1 (0.7)	Dhaun et al. [86]
CKD (non-transplant, no dialysis)	both	47 (10)	111	5.5(1.1)^*^	n.r.		3.0 (3.1)^*^	
Healthy Volunteers	Men	47(14)	8	4.2 (1.2)	12.52 (4.3)	ng/day	3.5 (2.0)	Goddard et al. [87]
CKD stage 2-5	Men	46(13)	8	5.6 (1.7)^*^	30.67 (13.8)^*^	ng/day	15.2 (11.7)^*^	
Controls	both	45 (3)	19	1.1 (0.1)	109 (21)	ng/day		Hoffman et al. [88]
Hypertensives	both	52 (4)	17	1.3 (0.3)^*^	29 (3)^*^	ng/day		
Salt resistant			7	n.r.	36 (5)	ng/day		
Salt sensitive			10	n.r.	23 (3)	ng/day		
Controls	both	53 (6)	12	0.6 (0.1)	n.r.			De Mattia et al. [49]
Lean, NNIDD, without microalbuminuria	both	50 (8)	18	1.59 (0.14)^*^	n.r.			
Lean, NNIDD, with microalbuminuria	both	49 (12)	9	1.97 (0.58)^*^	n.r.			
Lean, hypertensive†, without albuminuria	both	52 (3)	12	0.91 (0.19)^*^	n.r.			
Lean, hypertensive^†^, with albuminuria	both	52 (7)	10	1.4 (0.21)^*^	n.r.			
Controls	both	47 (12)	11	2.8 (0.8)	98.9 (24.7)	ng/day		Hwang et al. [48]
Hypertensives	both	48 (10)	23	2.0 (0.9)	86.0 (22.4)	ng/day		

NNIDD, normotensive, non-insulin-dependent diabetes, n.r., not reported.

* *P*<0.05 compared with the control group within that study.

† These hypertensive patients were without any prior anti-hypertensive treatment.

### Rat

**Figure 4:**
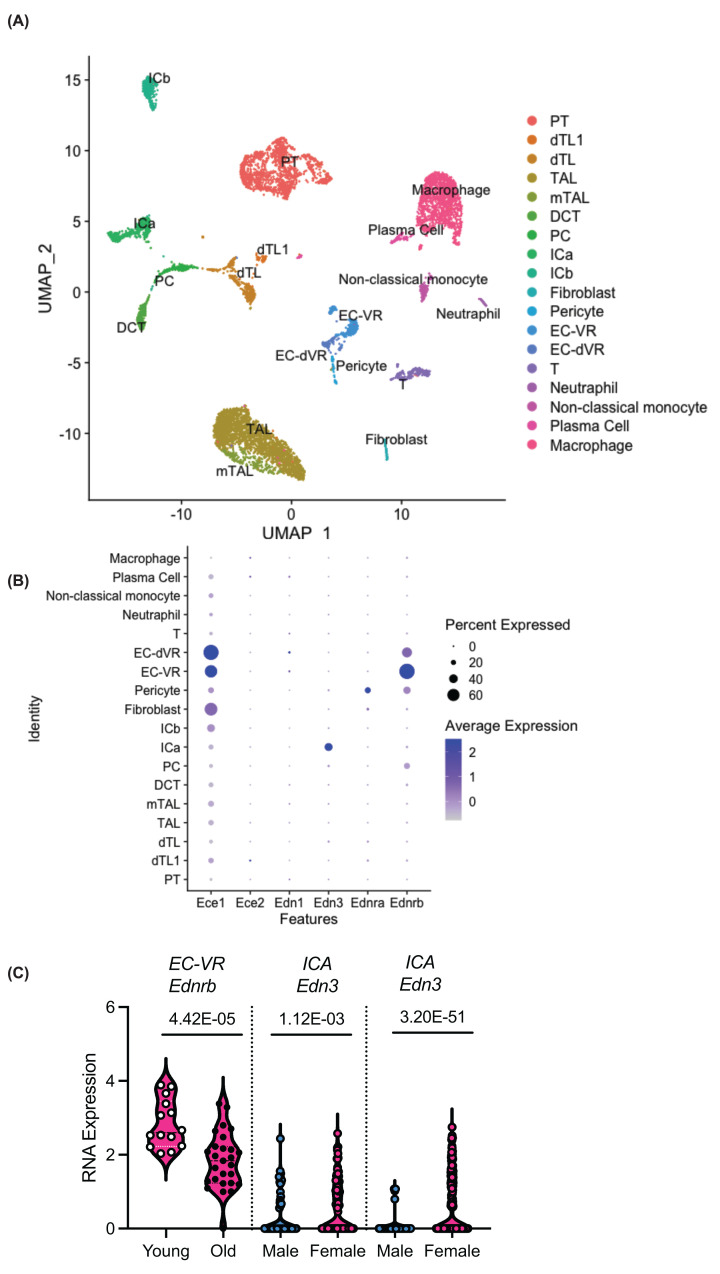
Unsupervised clustering of kidney single cells from male and female rats published by [37] A total of 8051 cells were RNA sequenced from a young female (5 months old), and aged female (27 months old), a young male (5 months) and aged male (27 months). (**B**) Dotplot of the RNA expression of the endothelin related genes across the clusters. (**C**) The only differentially expressed genes related to the endothelin system in this data set. *P*-values in the figure are adjusted for multiple comparisons. PT, proximal tubule; dTL1, descending thin limb-1; dTL, descending thin limb; TAL, thick ascending limb; mTAL, medullary thick ascending limb; DCT, distal convoluted tubule; PC, principal cell; ICA, intercalated cells alpha; ICB, intercalated cell β; EC-VR, endothelial cells-vasa recta; EC-dVR, endothelial cell descending vasa recta; T, T cell.

The rat scRNA-seq contained 8051 cells sequenced from the young and aged rats (Figure 4A) [37]. In addition to the epithelial, endothelial, and fibroblast cells we identified a large cluster of macrophages (Figure 4A). Plasma cells, neutrophils and non-classical monocytes were also identified. In agreement with human data, there was a high expression of *Ece1* and *Ednrb* in the endothelium, but low detection of *Ece2, Edn1, Edn3* (except ∼40% of intercalated-alpha cells (ICA) had high expression of *Edn3*), and *Ednra* (Figure 4B). There were only 3 DEGs in the rat dataset (Figure 4C). Between the sexes, male rat ICA cells have fewer *Edn3* transcripts than young females, and this difference persisted with age (Figure 4C). In the female rats, young ones had 2.7-fold higher *Ednrb* in the endothelium of the vasa recta (EC-VR) than aged females (Figure 4C).

**Figure 5:**
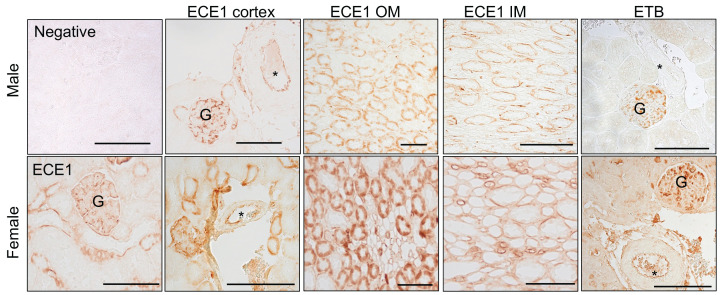
Representative images of rat kidneys use in immunolocalization of the ET system The negative control lacked the primary antibody. The ECE1 was expressed in the glomeruli (G), endothelium of blood vessels (*), thick ascending limbs, vasa recta and inner medullary (IM) collecting ducts. The ETB was abundant in the glomerulus and endothelium of the large kidney blood vessels of female rats. The scale bar represents 100 microns.

Immunohistochemical analysis of kidney sections from 10- to 12-week-old rats localized ECE1 in the endothelium, including the glomerular endothelium, and in the thick ascending limb (Figure 5). In the inner medulla, ECE1 was localized to the vasa recta and collecting ducts (Figure 5). In addition, ETB was highly expressed in the endothelium of the glomeruli of both sexes, while endothelial ETB expression in the kidney vessels was only immunolocalized in the female (Figure 5).

Plasma ET-1 and urinary ET-1 excretion in rats from on different salt diets from the literature are consolidated in Table 3. In Sprague Dawley rats, when acclimated to a high salt diet (8% NaCl) for months, plasma ET-1 was significantly greater than rats on a normal salt diet [50] (Table 3). Urinary ET-1 excretion was significantly greater in male rats acclimated to high salt diets (4−10%) for 5−14 days [51,52]; female rats had higher urinary ET-1 excretion than males on a normal salt diet and it was not further enhanced by acclimation to a high salt diet [51] (Table 3).

**Table 3:** Plasma ET-1 and urinary ET-1 excretion from rats and mice on different salt diets (mean [s.e.m.])

Species	Sex	Diet NaCl %	*n*	Plasma ET-1 pg/ml	Urinary ET-1 Excretion	Excretion units	Reference
Sprague Dawley	Male	0.4	5−8	n.r.	5.6(0.8)	pg/day/kg	Gohar et al. [51]
	Male	4.0	5−8	n.r.	10.6(0.7)	pg/day/kg	
	Female	0.4	6−11	n.r.	11.8(3.9)	pg/day/kg	
	Female	4.0	6−11	n.r.	14.9(2.3)	pg/day/kg	
Sprague Dawley	Male	0.64	8	3.5 (0.5)	n.r		Cordaillat et al. [50]
	Male	8.0	8	6.9 (0.4)^*^	n.r.		
Sprague Dawley	Male	0.8	6	∼0.55 (0.1)	2.3 (0.1)	ng/day	Sasser et al. [52]
	Male	10.0	6	∼0.6 (0.1)	6.7 (0.4)^*^	ng/day	
C57Bl6 Mice	Male	0.49	10	n.r.	0.08 (0.01)	pg/day	Guthrie et al. [53]
	Female	0.49	10	n.r.	0.17(0.03)^*^	pg/day	
C57Bl6 Mice							Wang et al. [55]
Sham	Male	1.0	6	1.6 (0.3)	n.r.		
5/6th nephrectomy	Male	1.0	6	3.4(0.3)^*^	n.r.		
C57Bl6	Male	0.49	6	n.r.	∼0.25 (0.01)	pg/day	Douma et al. [54]
	Male	4.0	5	n.r.	∼0.4 (0.01)	pg/day	
C57Bl6 xFVB	Male	0.8	9	0.31 (0.03)	n.r.		Saurage et al. [89]
C57Bl6 xFVB	Female	0.8	9	0.39 (0.07)	n.r.		
Mixed background	Both	0.8	5-9	5.3 (0.8)	16.2 (2.8)	pg/day	Ahn et al. [13]
	Both	2.7	5-9	5.0 (1.2)	31.2 (4.1)^*^	pg/day	

n.r., not reported, ∼ estimated from figures in the citation.

* *P*<0.05 compared with the control group or between the sexes within that study.

### Mouse

**Figure 6:**
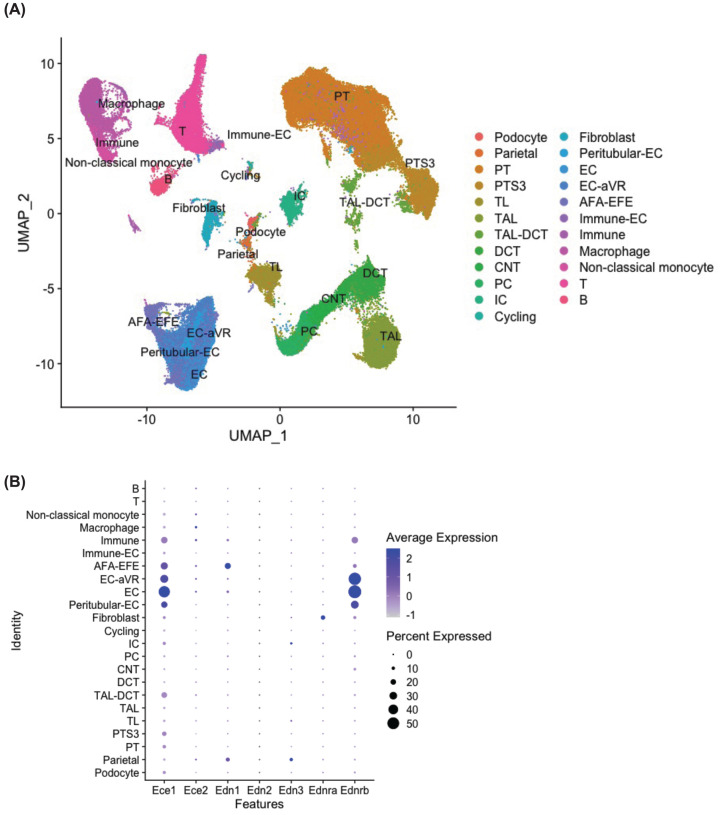
Unsupervised clustering of kidney single cells and nuclei from male and female mice (**A**) Unsupervised clustering of 99588 kidney single cells and single nuclei RNA sequenced from C57bl/6J male and female mice from [30,38,40–46]. (**B**) Dotplot of the RNA expression of the endothelin related genes across the clusters.

Using mouse datasets, we compiled and analyzed 99,588 cells/nuclei (Figure 6A). We found clusters of the expected cell types of the kidney, as well as group of cells with a high expression of cell cycle genes (cluster annotated at cycling). Consistently with the other species, *Ece1* and *Ednrb* were highly expressed in the murine endothelial clusters, and there was low detection of *Ece2, Edn1, Edn2, Edn2*, and *Ednra* (Figure 6B).

**Figure 7:**
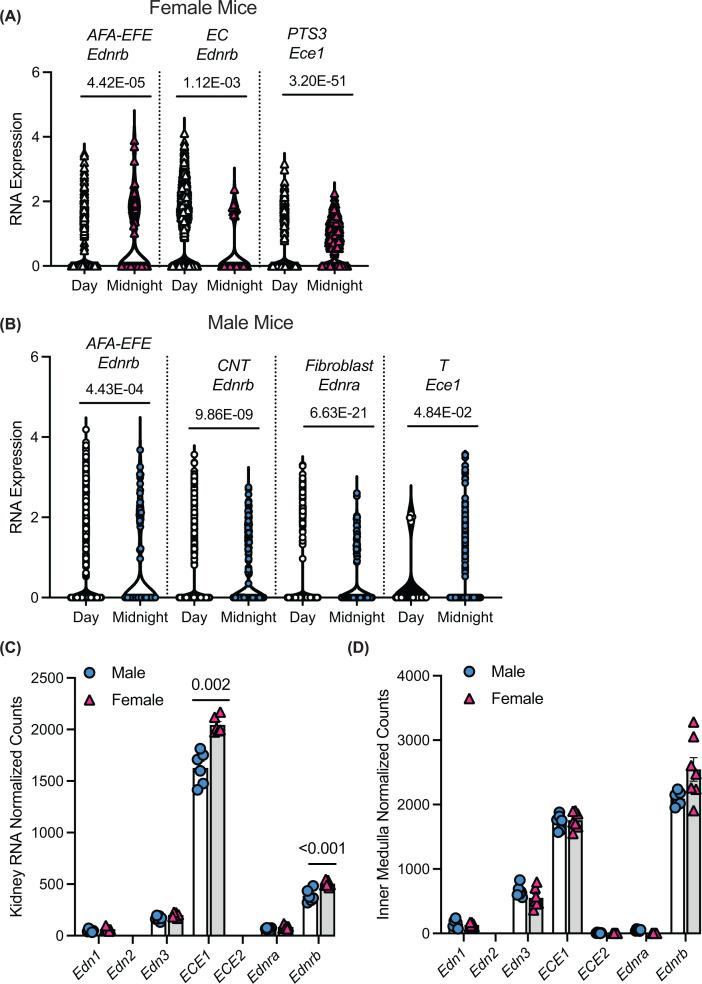
Violin plots of the normalized RNA expression of genes Violin plots of the normalized RNA expression of genes at the individual cells/nuclei level comparing day and midnight samples, for (**A**) female mice and (**B**) male mice. *P* values are reported above the plots for each pair and are adjusted *P* values for multiple comparisons. (**C,D**) Normalized counts of RNA from (C) whole mouse kidney or (D) mouse inner medulla. *P* values reported are adjusted for multiple comparisons. Individual animals are plotted. Blue circles represent male mice and pink triangles female mice. AFA-EFE, afferent-efferent arteriole; EC, endothelial cell; PTS3, proximal tubule segment 3; CNT, connecting tubule; T, T cell.

Sexually dimorphic DEGs included male mice having 20% fewer *Ednrb* transcripts in the endothelial cell (EC) and 30% fewer *Ednra* transcripts in the fibroblast than female mice (*P* = 3.03E-53, *P* = 3.36E-18, respectively). One unique aspect of this combined dataset is that we were able to determine diurnal differences in kidney single cell/nucleus transcriptomes (Figure 7). In the female kidneys, there were significant diurnal expression patterns of *Ednrb*; in the EC cluster *Ednrb* was greatest during the daytime, while in the afferent-efferent arteriole (AFA-EFE) cluster *Ednrb* was greatest at midnight (Figure 7A). In male mice, AFA-EFE *Ednrb* was also greatest at midnight as was connecting tubule (CNT) *Ednrb*, and fibroblast *Ednra* (Figure 7B). Interestingly, only in male kidneys *Ece1* in the T-cell cluster was significantly greater at midnight (Figure 7B).

Using our previously published murine whole kidney [38] and inner medulla bulk RNA-seq datasets [47] we determined normalized counts for the ET system (Figure 7C,D). In the whole kidney, *Ece1* and *Ednrb* were significantly greater in the female kidney compared with male kidney (Figure 7C), while no statistically significant differences in the normalized counts of any of the ET related genes were observed in the inner medulla (Figure 7D).

**Figure 8:**
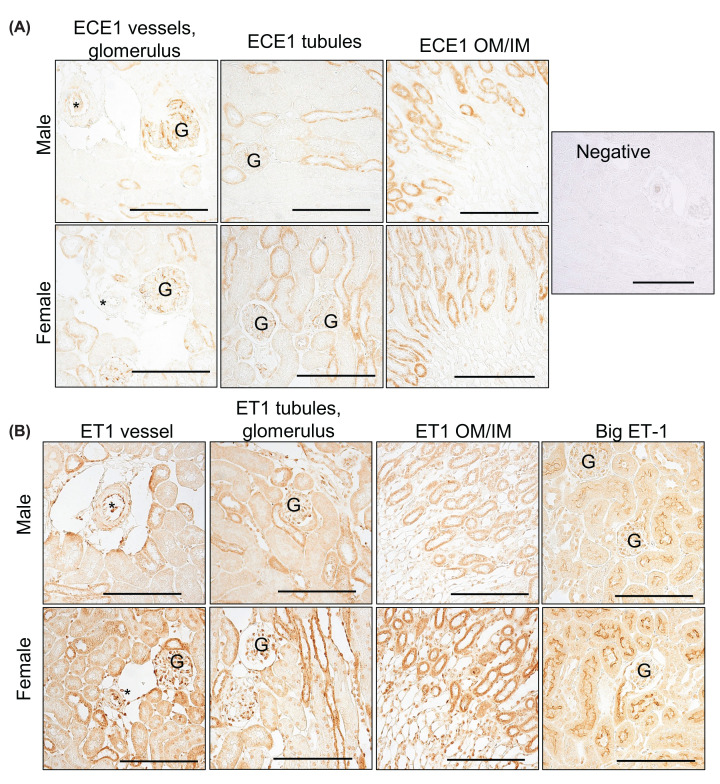
Representative images of mouse kidneys Representative images of mouse kidneys collected during the day time and used to immunolocalize the endothelin converting enzyme (ECE1), endothelin-1 (ET1), and big ET-1 of the ET system. (**A**) ECE1 was abundant in the glomerulus, and thick ascending limbs. Outer medulla, OM; Inner medulla, IM. Negative control lacked primary antibody. Asterisk (*) marks blood vessels. (**B**) ET-1 was abundant in the kidney blood vessels, glomerulus, and thick ascending limbs. There was also ET-1 found in interstitial cells. Big ET-1 was highly expressed in the S1 and S2 segments of the proximal tubules. Scale bar represents 100 microns.

ECE1 was immunolocalized to the endothelium of the glomerulus, with a minimal expression in the larger kidney vessels, regardless of sex (Figure 8A). Additionally, ECE1 was abundant in the thick ascending limbs of both sexes (Figure 8A). ET1 was detected in the cortical endothelium of large vessels and glomeruli (Figure 8B). Like ECE1, ET-1 was also abundant in the thick ascending limbs (Figure 8B). Conversely, Big-ET1 was observed in the brush border of the proximal tubules and selective for the S1 and S2 segments (Figure 8B).

Plasma ET-1 and urinary ET-1 excretion from various groups of mice are reported in Table 3. Like the Sprague Dawley rats, C57bl6/J female mice excreted more urinary ET-1 than male mice on a normal salt diet [53] (Table 3). When acclimated to a high salt diet (2.7% to 4.0% NaCl) male mice excreted more ET-1 than normal salt controls [13,54] (Table 3). Although plasma ET-1 concentration was similar between the sexes (Table 3), in male mice with reduced renal mass (5/6th nephrectomy model), plasma ET-1 concentration was greater than sham operated mice [55] (Table 3).

## Discussion

In the present study, we compiled over 200,000 kidney single cell/nucleus transcriptomes from mice, rats, and human kidney samples. Surprisingly, the *EDN1/Edn1* transcripts had a very low detection in this system, even though ET-1 is highly produced by the kidney [56–58], and we were able to immunolocalize it in the endothelium and tubules of the human and mouse. These findings can be explained by either the technology limitation of low capture efficiency of the single cell/nucleus workflow [59] or that it truly represents the biology of the *EDN1/Edn1* transcript (e.g., low expression). *Edn1* transcripts from the bulk mouse whole kidney or inner medullary RNA-seq were detectable, albeit at a low normalized counts. Thus, an important limitation to the single cell/nucleus platform is that lowly expressed genes may not be captured by the bead/bar code system, and it is likely that *EDN1/Edn1* is one of these genes. However, we could immunolocalize ET-1 in the human kidney in the endothelium, glomeruli, and tubules, confirming prior studies [58] and, as we consolidated, there are many studies reporting increased plasma ET-1 and/or urinary ET-1 excretion in disease states like CKD, diabetes, and hypertension. This would suggest that ET-1 protein levels or release and/or ET receptor activity may be more informative about ET-1 mediated signaling, rather than transcriptional expression.

Biopsies from women and men, and kidney sections from male and female mice showed similar ET-1 localization patterns. However, qualitatively, female mice had a greater intensity of epithelial ET-1 immunoreactivity and this agrees with previously published studies which demonstrated that kidney ET-1 production is enhanced in female rats and women [51]. It is hypothesized that in female/women greater kidney ET-1 promotes efficient return to sodium balance and buffers blood pressure in response to high dietary salt intake [51,60,61].

*ECE1/Ece1* was highly expressed in the human, rat, and mouse kidney single cell/nucleus endothelial cells, including glomerular endothelial cells and peritubular endothelial cells. It was also highly expressed in the mouse bulk kidney RNA datasets, where in the whole kidney, female mice had a greater *Ece1* expression than male mice. It was the only protein that was immunolocalized in the vascular endothelium and glomerulus of the human, rat, and mouse kidney sections. ECE1 localization was also similar between the sexes. In the rats and mice regardless of sex, ECE1 was abundant in the thick ascending limbs, collecting ducts, and vasa recta. Previous examinations of human (both sexes) biopsies reported immunoreactivity of ECE1 in the endothelium of kidney vessels and glomeruli and collecting ducts, but weak immunoreactivity to proximal tubules and distal tubules [62]. Thus, for ET-related genes with high expression, single cell/nucleus and antibody-mediated detection systems work well within the kidney of different species.

The *Edn2* gene was not detected in the mouse or rat RNA datasets, nor immunolocalized in their kidneys. However, in the human kidney, there was *EDN2* detected. Moreover, in the human kidney ET-2 was immunolocalized to the intra-renal branched renal arteries and vessels no smaller than the interlobar arteries in the kidneys from both sexes. Previous studies determine that in human kidney cortical and medullary samples, and in some human renal blood vessels (3/8 tested) *EDN2* is expressed (determine by quantitative real-time PCR [58], but it could not be immunolocalized nor the protein measured in kidney lysates by High Pressure Liquid Chromatography/radioimmunoassay [57]. Although the sex/gender of the kidney samples profiled were not reported [57,58] at least in normal healthy kidney there appears to be a small amount of ET-2 protein. In the context of this species dependent differences in ET-2, rodent models may not present as useful tools for ET-2 studies, and other experimental models are needed to elucidate ET-2 mediated mechanisms.

Single-nucleotide polymorphisms near the *EDN3* gene have been associated with blood pressure regulation [63,64], and reduced risk of hospitalized bacteremia events in end-stage renal disease patients [65]. However, the *EDN3/Edn3* gene has low expression in the kidney. In the human kidneys used in our study, the *EDN3* transcript and ET-3 were not detected, although a previous study with quantitative PCR did detect *EDN3* [58] but failed to detect the protein [57]. However, in the rat and mouse single cell/nucleus RNA-seq *Edn3* was detected in intercalated cells. Moreover, there was greater intercalated cell *Edn3* in female rats compared with male rats, regardless of age. ET-3 protein was measured in kidneys from female Wistar rats where the greatest abundance of ET-3 was in the medulla of 33-month-old female rats [66]. Although at the kidney bulk RNA-seq level, we did not detect a significant sex difference in *Edn3* (*P*=0.11), in a mouse hypertensive model (Schlager BPH/2J) and their normotensive control (BPN/3J), females had a 1.7- to 1.9-fold increase in kidney *Edn3* [67]. Unfortunately, the commercially available anti-ET-3 that we used in our study failed to immunolocalize ET-3 in any kidney section from human, mouse, or rat. Also there appears to be no reports of ET-3 immunolocalization or *in situ* hybridization in the kidney of any species. Thus, the identification of *Edn3* in the intercalated cells is novel and warrants further evaluation on the protein level.

Binding sites for ET1, ET2, and ET3 were discovered in the kidney using radio-ligand studies. In human kidney sections, there was a high density of ET-receptor binding in the medulla and glomeruli [68–71]. Likewise in the rat kidney the medulla and glomeruli have a high density of ET-receptor binding [71,72]. We now know that there are 2 ET receptors in mammals, ETA [73] and ETB [74] with expression of both throughout the kidney. In the human kidney single cell RNA-seq dataset we found high expression of *EDNRA* in the kidney vascular smooth muscle cells, fibroblasts, myofibroblast, and renin positive cells (likely the juxtaglomerular apparatus cells). *EDNRB* was highly expressed also in the renin positive cells, but also the principal cells, and endothelium. In living donor kidney samples, men had greater glomerular endothelial cell *EDNRB* than women. When comparing within a sex, AKI and CKD kidneys had fewer *EDNRB* transcripts in degenerative epithelia, principal cells (in women), and glomerular endothelial cells (men). We were unable to immunolocalize these receptors in the kidney biopsies sections to confirm if there are sex differences of the receptors at the protein level. However, previous studies have demonstrated that there are sex-specific distributions of the ET receptors throughout the body. For example, middle-aged and older men have greater ETA-mediated vasoconstriction in the forearm compared with age-matched women [75]. In the saphenous vein (collected from individuals undergoing coronary artery bypass graft surgery), women had fewer ET receptors than men and the ratio of ETA:ETB was 50:50 compared with that of the men 75:25 [76]. In young women, ETB-dependent microvascular dilation is dependent on estradiol [77], and this ETB function is loss in postmenopausal women [78], or in women with polycystic ovary syndrome with excess androgens [79]. Whether there are sex-dependent differences in the kidney ET receptor abundance remains to be determined in humans.

Similar expression patterns for the ETA and ETB were found in the rat and mouse kidneys, with *Ednra* highly expressed in pericytes and fibroblasts, while *Ednrb* was enriched in the endothelium. In female rats, young ones had greater endothelial cell *Ednrb* than aged. In mice, females had greater endothelial cell *Ednrb* than males and this sex difference was observed at the protein level in the endothelium of the large kidney vessels of rats. There are many examples of ET receptor density and function differences in rodents that were recently reviewed [80]. There were also statistically significant effects on the receptors given the time of day. In male and female mice, afferent-efferent arteriole *Ednrb* was greatest at midnight. In females all other endothelial cells had greater *Ednrb* expression during the daytime. In male mice, *Ednra* was greatest in fibroblasts at midnight, and connecting tubule *Ednrb* was greatest at midnight. We were unable to immunolocalize the receptors in our mouse kidneys. If these time-of-day changes in receptor RNA correlate with protein or functional outcomes remains to be determined. However, previously reported studies suggest potential functional differences, as ET-1 excretion is higher during the active period in rats [51,81]. Surprisingly, *Ednrb* had low detection in the rodent principal cells, even though it is established that the inner medullary collecting ducts, which are primarily composed of principal cells [82], have a high density of receptors for ET-1 [83,84]. It is possible that these principal cells represent a greater proportion of cortical principal cells than inner medullary, or that the capture efficiency of the epithelial *Ednrb* is low with this technology in rodents.

In conclusion, reliable tools to detect the kidney endothelin system within a species or among different species remain a challenge. All approaches have limitations and although single cell/nucleus RNA-sequencing (or other omics approaches like chromatin accessibility) are attractive, they may not efficiently capture lowly expressed genes, like *EDN1/Edn1*. But as technology advances, and more sensitive tools are developed, this will have a great benefit on the ET system research. Although there are strengths to using monoclonal antibodies, such as a homogenous antibody population thus limiting batch effects, in our hands, few seemed to immunolocalize in the kidney. Thus, there is a need to develop better tools to integrate the ET system. Until that is achieved multiple approaches should be considered when evaluating the intrarenal ET system to overcome the technical limitations and provide rigorous and reproducible data.

## Supplementary Material

Supplementary Tables S1-S3

## Data Availability

All single cell and nucleus RNA sequencing data is deposited in GEO and in the KPMP as reported in the methods.

## Abbreviations

AKI: acute kidney injury
CKD: chronic kidney disease
ECE1: endothelin-converting enzyme-1
ERA: endothelin receptor antagonist
ET: endothelin
LD: living donor
