# Supplementary material for: Comprehensive analysis of the endothelin system in the kidneys of mice, rats, and humans
Source: Biosci Rep. 2024 Jul 12;44(7):BSR20240768. doi: 10.1042/BSR20240768 (PMC11249498; doi:10.1042/BSR20240768)
Supplement: Supplementary Tables S1-S3 [file BSR-2024-0768_supp.pdf]

Comprehensive analysis of the endothelin system in the kidneys of mice, rats, and humans.

Margi Patel[1,#], Nicholas Harris[1,#], Malgorzata Kasztan[2], Kelly A. Hyndman[1]*

[1]University of Alabama at Birmingham, Department of Medicine, Division of Nephrology, Section of Cardio-Renal Physiology and Medicine, Birmingham, AL 35233

[2] University of Alabama at Birmingham, Department of Pediatrics, Division of Hematology-Oncology, Section of Cardio-Renal Physiology and Medicine

Supplementary Tables

Supplementary Table 1: Demographics of the subjects where the kidney biopsies for the immunohistochemistry were obtained. These biopsies were obtained from the Cooperative Human Tissue Network.

**Sample size**	9
**Gender, Male/Female**	4/5
**Age, years**	
20-29	1
30-39	0
40-49	0
50-59	3
60-69	3
>70	2
**Race**	
White	8
Black	1
Asian	0
**Collected from**	
Autopsy	2
Surgery	7
**Other information**	
Renal Cell Carcinoma	4
kidney cysts	1
splenic rupture	1*
liposarcoma	1
hydronephrosis	1
hypertension history	1*

* Indicates samples from autopsy

Supplementary Table 2: Demographics of the individuals who provided the samples for the Kidney Precision Medicine Project single cell RNA-sequencing. Data are reported as percents except for the sample size and gender distribution.

	**Living Donor**	**AKI**	**DKD***	**HCKD***
Sample Size	18	12	12	3
**Gender, Male/Female**	39/72	75/25	25/75	66/33
**Age, years**				
<30	0.00	0.00	0.00	0.00
30-39	33.33	41.67	16.67	0.00
40-49	33.33	83.33	0.00	0.00
50-59	33.33	125.00	8.33	33.33
60-69	5.56	41.67	41.67	66.67
70-79	0.00	0.00	33.33	0.00
**Race**			0.00	
White	n.r.	58.33	58.33	100.00
Black	n.r.	41.67	25.00	0.00
Asian	n.r.	0.00	8.33	0.00
Other		0.00	8.33	0.00
**AKI Stage, KDIGO**				
Stage 1		16.67		
Stage 2		25.00		
Stage 3		58.33		
**Baseline eGFR**				
20-29 ml/min/1.73m2	n.r.	0.00	8.33	33.33
30-39 ml/min/1.73m2	n.r.	0.00	16.67	0.00
40-49 ml/min/1.73m2	n.r.	0.00	41.67	33.33
50-59 ml/min/1.73m2	n.r.	8.33	0.00	33.33
>60-69 ml/min/1.73m2	n.r.	91.67	33.33	0.00
**Proteinuria or albuminuria**	n.r.	50.00	91.67	66.67
**Proteinuria unknown**		50.00	8.33	33.33
**History of diabetes**	n.r.	41.67	100.00	33.33
**History of hypertension**	n.r.	33.33	91.67	100.00
**On RAAS Blockade**	n.r.	33.33	58.33	66.67

AKI- acute kidney injury, DKD- diabetic kidney disease, HCKD- hypertensive chronic kidney disease, n.r. – not reported or available to us.

*note in the manuscript we call the DKD/HCKD collectively CKD.

Supplementary Table 3: All abbreviations of the cell types and their counts in the human single cell RNA-sequencing analyses from the Kidney Precision Medicine Project.

		All Groups	Living Donor		AKI		CKD	
**Cell**	**Name**	**Total**	**Men**	**Women**	**Men**	**Women**	**Men**	**Women**
POD	Podocyte	**244**	35	83	39	5	3	79
PEC	Parietal Epithelial Cell	**631**	80	78	161	44	39	229
PTS1S2	Proximal Tubule Epithelial Cell Segment 1&2	**7041**	1172	2560	159	58	443	2649
PT-S3	Proximal Tubule Epithelial Cell Segment 3	**181**	2	20	69	7	8	75
aPT	Proximal Tubule Epithelial Cell (adaptive / maladaptive / repairing1)	**9878**	393	641	2315	911	2045	3573
dPT	Proximal Tubule Epithelial Cell (degenerative3)	**5787**	51	103	2709	876	307	1741
dPT-DTL	Proximal Tubule Epithelial Cell / Descending Thin Limb Cell (degenerative3)	**2423**	60	154	822	181	133	1073
DTL1	Descending Thin Limb Cell Type 1	**1719**	16	41	106	770	67	719
M-TAL	Medullary Thick Ascending Limb Cell	**1074**	11	30	414	20	149	450
aTAL1	Thick Ascending Limb Cell Cluster 1 (adaptive / maladaptive / repairing1)	**1023**	2	1	533	231	23	233
aTAL2	Thick Ascending Limb Cell Cluster 2 (adaptive / maladaptive / repairing1)	**6629**	98	187	1924	584	798	3038
dC-TAL	Cortical Thick Ascending Limb Cell (degenerative3)	**1923**	68	49	920	81	166	639
C-TAL	Cortical Thick Ascending Limb Cell	**15850**	1349	2122	4390	556	2237	5196

REN	Renin-positive Juxtaglomerular Granular Cell	**168**	27	57	10	4	30	40
DCT1	Distal Convoluted Tubule Cell Type 1	**3289**	416	1197	86	53	369	1168
dDCT	Distal Convoluted Tubule Cell (degenerative3)	**1600**	9	25	618	94	131	723
CNT	Connecting Tubule Cell	**3217**	337	1194	226	31	324	1105
CNT-PC	Connecting Tubule Principal Cell	**549**	105	63	26	41	49	265
CNT-IC-A	Connecting Tubule Intercalated Cell Type A	**1988**	184	250	345	67	261	881
dCNT	Connecting Tubule Cell (degenerative3)	**1864**	2	23	1113	357	30	339
dCNT-PC	Connecting Tubule cell-Principal cell (degenerative3)	**3125**	73	63	215	14	2530	230
PC	Principal cell	**4801**	363	727	655	297	441	2318
dPC	Principal cell (degenerative3)	**2335**	51	65	449	276	379	1115
tPC-IC	Principal-Intercalated Cell (transitional4)	**1687**	64	454	83	1	720	365
IC-A	Intercalated Cell Type A	**3352**	336	734	360	134	264	1524
IC-B	Intercalated Cell Type B	**673**	74	136	112	27	44	280
dIC-A	Intercalated Cell Type A (degenerative3)	**429**	28	32	77	14	14	264
dEC-PTC	Peritubular Capillary Endothelial Cell (degenerative3)	**262**	11	20	45	30	36	120
EC-AEA	Afferent / Efferent Arteriole Endothelial Cell	**1748**	89	185	423	93	169	789
EC-PTC	Peritubular Capillary Endothelial Cell	**4229**	407	1073	691	236	414	1408
EC-GC	Glomerular Capillary Endothelial Cell	**1426**	206	564	155	23	105	373
cycEC	Endothelial Cell (cycling2)	**39**	1	3	12	2	3	18
EC-LYM	Lymphatic Endothelial Cell	**105**	2	10	16	29	8	40

dVSMC	Vascular Smooth Muscle Cell (degenerative3)	**849**	72	180	191	39	70	297
VSMC-P	Vascular Smooth Muscle Cell / Pericyte	**1228**	131	371	244	42	116	324
MC	Mesangial Cell	**130**	3	39	31	12	3	42
FIB	Fibroblast	**720**	40	145	194	25	92	224
aFIB	Fibroblast (adaptive / maladaptive / repairing1)	**178**	26	33	66	8	7	38
MyoF	Myofibroblast	**76**	3	5	25	5	4	34
MAST	Mast cell	**31**	0	0	2	4	0	25
NKT	Natural Killer T Cell	**1252**	9	27	807	82	87	240
NK1	Natural killer 1	**791**	57	79	340	86	39	190
NK2	Natural killer 2	**206**	4	5	55	38	14	90
PL	Plasma Cell	**406**	0	4	282	4	34	82
pDC	Plasmacytoid Dendritic Cell	**207**	1	3	109	37	5	52
cDC	Classical Dendritic Cell	**1708**	36	40	689	217	110	616
B	B Cell	**1148**	23	92	445	88	99	401
MON	monocyte	**1329**	19	53	555	176	122	404
ncMON	Non-classical Monocyte	**367**	4	11	133	64	20	135
MAC-M2	M2-Macrophage	**892**	28	71	428	119	45	201
T	T Cell	**4359**	148	316	1368	639	172	1716
T-CYT	T cytotoxic	**947**	30	67	370	109	57	314
cycT	Tcell (cycling)	**232**	7	18	143	10	15	39
T-REG	T regs	**241**	3	4	94	56	14	70
MDC	Monocyte-derived Cell	**1332**	59	127	372	90	65	619
cycMNP	Mononuclear Phagocyte (cycling2)	**209**	0	0	111	32	7	58
cycEPI	Epithelial cell (cycling2)	**219**	1	3	128	38	8	42
**Sum**		**110346**	**6826**	**14637**	**27460**	**8167**	**13944**	**39312**
